# Severe everolimus-induced steatohepatis: a case report

**DOI:** 10.1186/2047-783X-18-22

**Published:** 2013-07-03

**Authors:** Gisela Schieren, Edwin Bölke, Axel Scherer, Andreas Raffel, Peter Arne Gerber, Patric Kröpil, Matthias Schott, Jackson Hamilton, Anne Hayman, Wolfram Trudo Knoefel, Wilfried Budach, Christiane Matuschek

**Affiliations:** 1 Department of Nephrology, Medical Faculty, Heinrich Heine University of Düsseldorf, Moorenstr. 5, 40225, Düsseldorf, Germany; 2 Department of Radiation Oncology, Medical Faculty, Heinrich Heine University of Düsseldorf, Düsseldorf, Germany; 3 Department of Diagnostic and Interventional Radiology, Luisenhospital Aachen, Aachen, Germany; 4 Department of General Surgery, Medical Faculty, Heinrich Heine University of Düsseldorf, Düsseldorf, Germany; 5 Department of Dermatology, Medical Faculty, Heinrich-Heine University Düsseldorf, Düsseldorf, Germany; 6 Department of Diagnostic and Interventional Radiology, University of Dusseldorf, Medical Faculty, Düsseldorf, Germany; 7 Department of Endocrinology, Medical Faculty, Heinrich Heine University of Düsseldorf, Düsseldorf, Germany; 8 Department of Diagnostic Radiology, The University of Texas MD Anderson Cancer Center, Houston, Texas, USA; 9 Department of Diagnostic Radiology, Baylor College of Medicine, Houston, Texas, USA

## Abstract

The mammalian target of rapamycin inhibitors are normally favored as immunosuppressant agents for solid organ transplantation such as kidney, liver or heart. Only in recent years have they been increasingly administered for the treatment of neuroendocrine tumors. Even though mammalian target of rapamycin inhibitors are known to exhibit specific side effects, everolimus-related severe hepatic steatosis has not as yet been described in the literature. We report the case of a 76-year-old man who developed severe hepatic steatosis within four weeks of treatment with everolimus as concomitant tumor therapy for a progressively growing neuroendocrine carcinoma of the ileum. A diagnosis of hepatic steatosis was established using computer tomography and fibroscan^©^. Other underlying causes for steatosis hepatis could be excluded. Further studies are warranted to explain the underlying mechanisms.

## Background

In recent years new cancer treatments have been developed [1,2]. Everolimus is an inhibitor of the mammalian target of rapamycin (mTOR) and has been approved for the prevention of organ transplant rejection as well as for the treatment of subependymal giant cell astrocytoma, neuroendocrine tumors, renal cell carcinoma and breast cancer [3-8]. mTOR inhibitors are increasingly administered for the treatment of neuroendocrine tumors [9,10]. The most frequent everolimus-associated adverse effects include myelosuppression, which may result in anemia, thrombo- and lymphocytopenia, and subsequent complications such as infections [4]. Non-infective pneumonitis, pleural or pericardial effusions, elevated transaminases, hyperlipidemia, electrolyte disorders, edema, hypertension, gastrointestinal symptoms and renal failure including proteinuria are also observed [11]. Here, we report a case of severe hepatic steatosis following everolimus therapy in a patient with a neuroendocrine tumor of the ileum.

## Case presentation

A 76-year-old male patient was diagnosed with a highly differentiated neuroendocrine carcinoma of the ileum (Ki67 15%) in November 2009, including liver metastases. In December 2009 he underwent a segmental resection of the ileum and multiple (n = 23) atypical liver resection (cherry picking) of segments II, III, IV, V, VI, VII and VIII. In January 2010 he was discharged from hospital, followed by a total of six monthly intravenous applications of octreotide (Sandostatin^©^; 3 × 0.2 mg subcutaneously). In July 2010, the patient developed a recurrence of his liver metastases. Selective internal radiotherapy was planned. After radiologic evaluation, the duodenal branches of the right and left hepatic artery were surgically divided because of difficult interventional access; selective internal radiotherapy treatment was then performed. A magnetic resonance tomography scan of his abdomen in October 2010 and a computed tomography scan of his abdomen and thorax in February 2011 showed no signs of local cancer recurrence or distant metastasis.

In June 2011, magnetic resonance tomography of the abdomen revealed a liver metastasis in segment V. In addition, multiple bone metastases (9. left costae, os sacrum, os ileum with iliosacral joint) were suspected on a whole body scintigraphy with 200 MBq indium-111-octreotide. The patient required surgery for a symptomatic incisional hernia. Because the most dynamic progression was seen in the liver, the liver lesions were resected again (cherry picking) during the repair of the incisional hernia. In August 2011, chemotherapy with streptozotocin (500 mg/m^2^ body surface) and 5-fluorouracil (500 mg/m^2^ body surface) was initiated. In September 2011 a port catheter system was inserted. One month later, after three cycles of streptozotocin, the patient developed aplasia accompanied by severe mucositis grade III (CTC- scale) and chemotherapy had to be interrupted. Nevertheless the tumor progressed further and the patient was switched to (DOTA0-Phe1-Tyr3)octreotide chemotherapy (also known as edotreotide) in January 2012. After the development of distant metastases in the thoracic and lumbar spine, a palliative radiotherapy of 30 Gy was administrated from thoracic vertebra 12 to lumbar vertebra 4 in April 2012. In June 2012, everolimus treatment was started (10 mg/day orally). Within four weeks the patient developed severe ascites, accompanied by steadily rising liver enzymes.

Before the start of everolimus treatment, the patient had moderately elevated but stable triglyceride blood levels (158 mg /dL) for many years. As he had a history of coronary artery disease, he was on simvastatin treatment and his cholesterol blood levels were stable around 120 mg/dL. Probably because of his liver metastases and repetitive atypical liver resections with compromised liver function, we observed low total protein levels (5.89 g/dL), low albumin levels (2.2 g/dL), elevated liver enzymes (alanine transaminase (ALT): 58 U/=L, aspartate transaminase (AST): 9 U/L, gamma glutamyl transpeptidase: 1.350 U/L), and an increased lactate dehydrogenase blood concentration (407 U/lL) shortly before mTOR inhibitor treatment was started. At that time direct bilirubin was within normal range (0.49 mg/dl). After four weeks of everolimus treatment, liver enzymes increased (maximal ALT: 16 U/L, AST: 169 U/L, gamma glutamyl transpeptidase: 1599 U/L, lactate dehydrogenase 571 U/L) while bilirubin levels stayed within normal range. Viral hepatitis could be excluded because hepatitis B surface antigen as well as hepatitis B, C and E virus serology were negative. There were no signs of diabetes: blood sugar tests, which had been routinely taken during his hospital stays as well as his visits to the outpatient clinics, were within normal range. Furthermore the patient had no signs of metabolic syndrome before receiving everolimus. At the start of everolimus treatment, his body mass index was 26.3 kg/m^2^ and within the normal range (height 170 cm, weight 75 kg). He did not lose or gain weight rapidly before starting everolimus. Except for everolimus, no other chemotherapeutic agent or new medication was administered that could cause a fatty liver.

**Figure 1:**
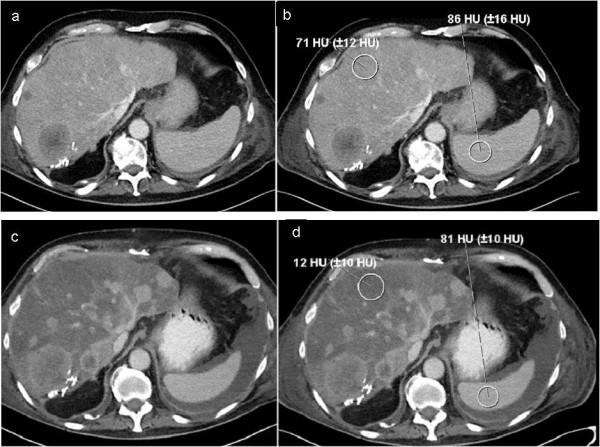
**Pre-therapy and follow-up computed tomography images. (a,b)** The pre-therapy axial post-contrast computed tomography images (with region of interest measurements) shows multiple liver metastases that were hypodense compared with the normal density of the liver and spleen parenchyma. **(c,d)** In the follow-up examination, a progress of the metastases was noted and a diffuse abnormal low density of the liver. HU, Hounsfield units.

Because of the increased liver enzymes and abdominal pain, computer tomography of the abdomen was performed and revealed severe hepatic steatosis (Figure 1). The diagnosis was confirmed by a fibroscan even though examination results were difficult to interpret due to difficult examination conditions (ascites and air). The hepatic steatosis developed rapidly - the histopathology of the specimen obtained from the atypical liver resection in 2009 had not revealed any signs of liver steatosis at that time. Even though everolimus treatment was stopped, the patient developed multiorgan failure eight weeks later and died.

## Conclusions

Even though everolimus has been described to cause hepatic adverse events, including an increase in AST (up to 25%), increased ALT (up to 21%) and increased bilirubin (up to 3%), a rapid onset of severe hepatic steatosis has not been reported in the literature. Causes of rapid steatosis hepatis may include diabetes mellitus, protein malnutrition, hypertension, obesity, rapid weight loss and sleep apnea. In addition, certain cell toxins such as oxygen radicals and certain drugs are associated with hepatic steatosis.

Our patient had a history of malnutrition. Nevertheless, total protein and albumin levels had been stable for months. A history of alcohol consumption was excluded. There was no history of diabetes mellitus. Cardiovascular disease had been stable for several years. The patient received none of the drugs usually associated with non-alcoholic fatty liver disease. Viral hepatitis was also excluded. As the rapid onset of the clinical features correlated strongly with the beginning of the everolimus treatment, we propose that hepatic steatosis in our patient was caused by everolimus.

One might speculate upon the mechanism by which everolimus could induce hepatic steatosis beside a direct toxic effect. Osawa *et al*. reported that rapamycin attenuated hepatocyte lipid accumulation induced by fatty acid with serotonin, suggesting a rather protective role of mTOR inhibition in this setting [12]. Molecular and cellular mechanisms of steatosis hepatis have not been fully elucidated, but insulin resistance has been discussed as a possible key mechanism. Chronic administration of the mTOR inhibitor rapamycin has been shown to substantially impair glucose tolerance and insulin action, inducing muscle insulin resistance despite weight loss in rats [13]. Lamming *et al*. demonstrated that this effect is probably mediated by disruption of a second mTOR complex, mTORC2, which is required for the insulin-mediated suppression of hepatic gluconeogenesis [14]. A similar mechanism may be responsible for the induction of steatosis hepatis in our patient. In this scenario, the pre-existing impaired liver function and malnutrition might have promoted the development of everolimus-induced steatosis hepatitis. Additional studies are needed to clarify the pathomechanism involved.

With regard to the steadily increasing indications for everolimus therapy, treating physicians should be aware of the potential adverse effect of rapid and severe steatosis hepatis in patients with pre-existing liver disease.

## Consent

Written informed consent was obtained from the patient for publication of this Case report and any accompanying images. A copy of the written consent is available for review by the Editor-in-Chief of this journal.

## Abbreviations

ALT: Alanine transaminase; AST: Aspartate transaminase; mTOR: Mammalian target of rapamycin.

## Competing interests

The authors declare that they have no competing interests.

## Authors’ contributions

All authors participated in the publication preparation and drafted the manuscript. GS collected all clinical data. All authors read and approved the final manuscript.
